# Toxic epidermal necrolysis-like linear IgA bullous dermatosis as a manifestation of multiple drug hypersensitivity in the setting of drug reaction with eosinophilia and systemic symptoms

**DOI:** 10.1016/j.jdcr.2024.04.028

**Published:** 2024-04-27

**Authors:** Scott Stratman, Lisa Zhou, Randie H. Kim, Robert G. Phelps, Jacob Glickman, Daniela Mikhaylov, Jianni Wu, Nour El-Kashlan, Ryan Rivera-Oyola, Jonas A. Adalsteinsson, Melissa A. Levoska, Nicholas Gulati

**Affiliations:** a Department of Dermatology, Icahn School of Medicine at Mount Sinai, New York, New York; b Dermpath Diagnostics, White Plains, New York

**Keywords:** corticosteroids, DRESS, drug-induced linear IgA disease, drug reaction, drug reaction with eosinophilia and systemic symptoms, LABD, linear IgA bullous dermatosis, meropenem, TEN, toxic epidermal necrolysis, toxic epidermal necrolysis-like linear IgA bullous dermatosis

## Introduction

Drug reaction with eosinophilia and systemic symptoms (DRESS) is a hypersensitivity reaction mediated by T cells. Many drug classes are implicated in DRESS, including antibiotics, antivirals, and anticonvulsants. Even after stopping the culprit drug, DRESS symptoms can persist for months.^1^ “Flare-up” reactions are exacerbations of *identical* drug hypersensitivity reaction symptoms when a new drug is given while the immune system is still up regulated by the initial hypersensitivity reaction, typically occurring within hours up to 48 hours after starting the new drug.^2^ In contrast, multiple drug hypersensitivity (MDH) presents as unique cutaneous symptoms to at least 2 chemically distinct substances.^2^, ^3^, ^4^ The initial manifestation of MDH can be erythroderma, exanthema or DRESS.^5^ In the context of MDH, subsequent cutaneous manifestations may be similar to or different from the first drug hypersensitivity reaction.^5^ Of particular concern, Stevens-Johnson syndrome/toxic epidermal necrolysis (TEN) can develop in the setting of MDH, typically manifesting in the second or third episode from a unique drug exposure. Linear IgA bullous dermatosis, however, rarely presents as a sequential manifestation in MDH.^5^ Idiopathic linear IgA bullous dermatosis classically presents as vesiculobullous lesions arranged in an annular pattern. Drug-induced linear IgA bullous dermatosis (DILABD) is typically more severe and may even mimic TEN with a positive Nikolsky sign.^6^^,^^7^ Herein, we discuss a patient with MDH who initially presented with DRESS and later developed Stevens-Johnson syndrome/TEN-like DILABD from 2 distinct antibiotic culprits.

## Case report

**Fig 1:**
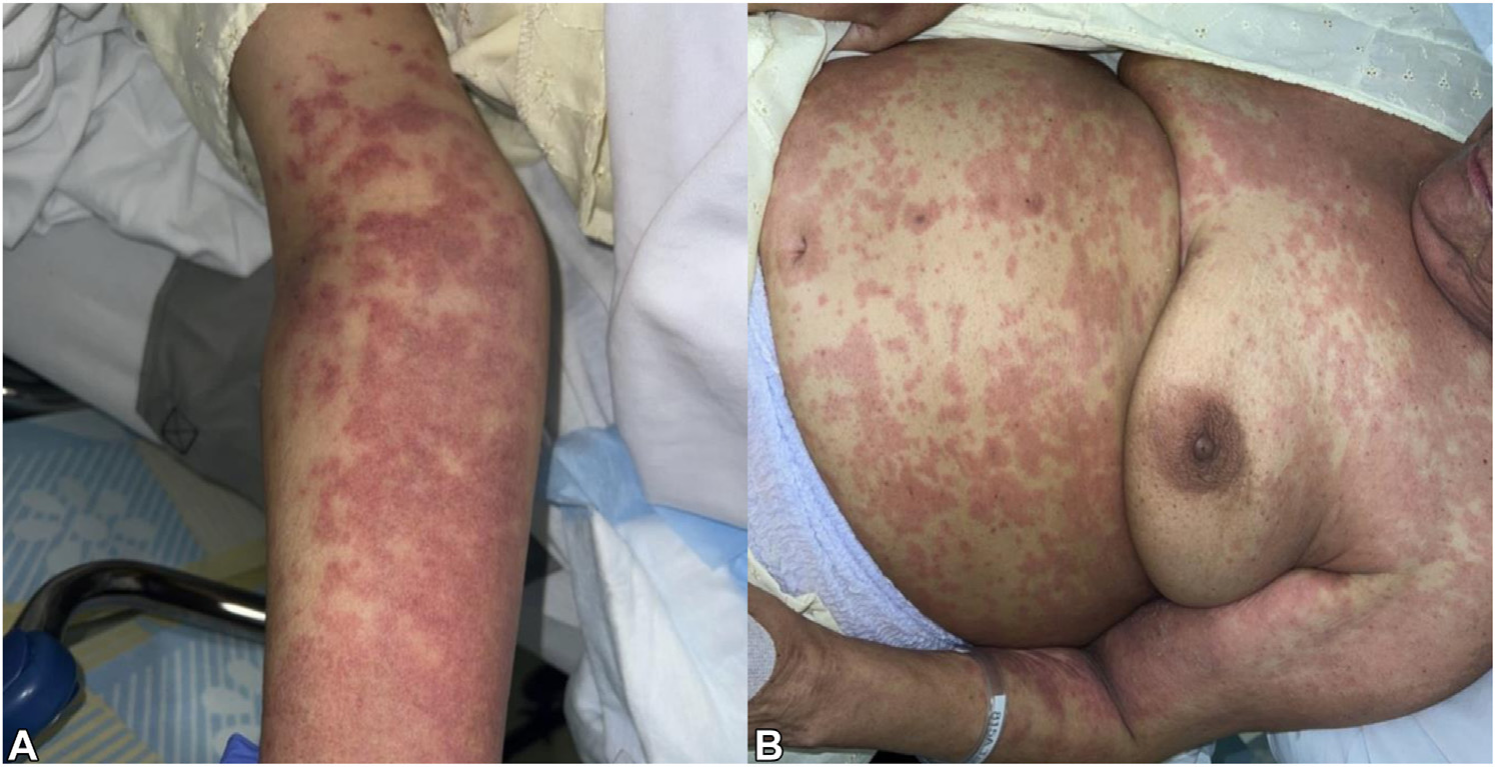
Initial presentation of morbilliform rash involving the (**A**) right upper extremity and (**B**) trunk which started 4 days after administration of isoniazid. Given eosinophilia, facial edema, elevated creatinine, and RegiSCAR score of 4, the patient’s condition was concerning for DRESS, and she was treated with oral steroids.

**Fig 2:**
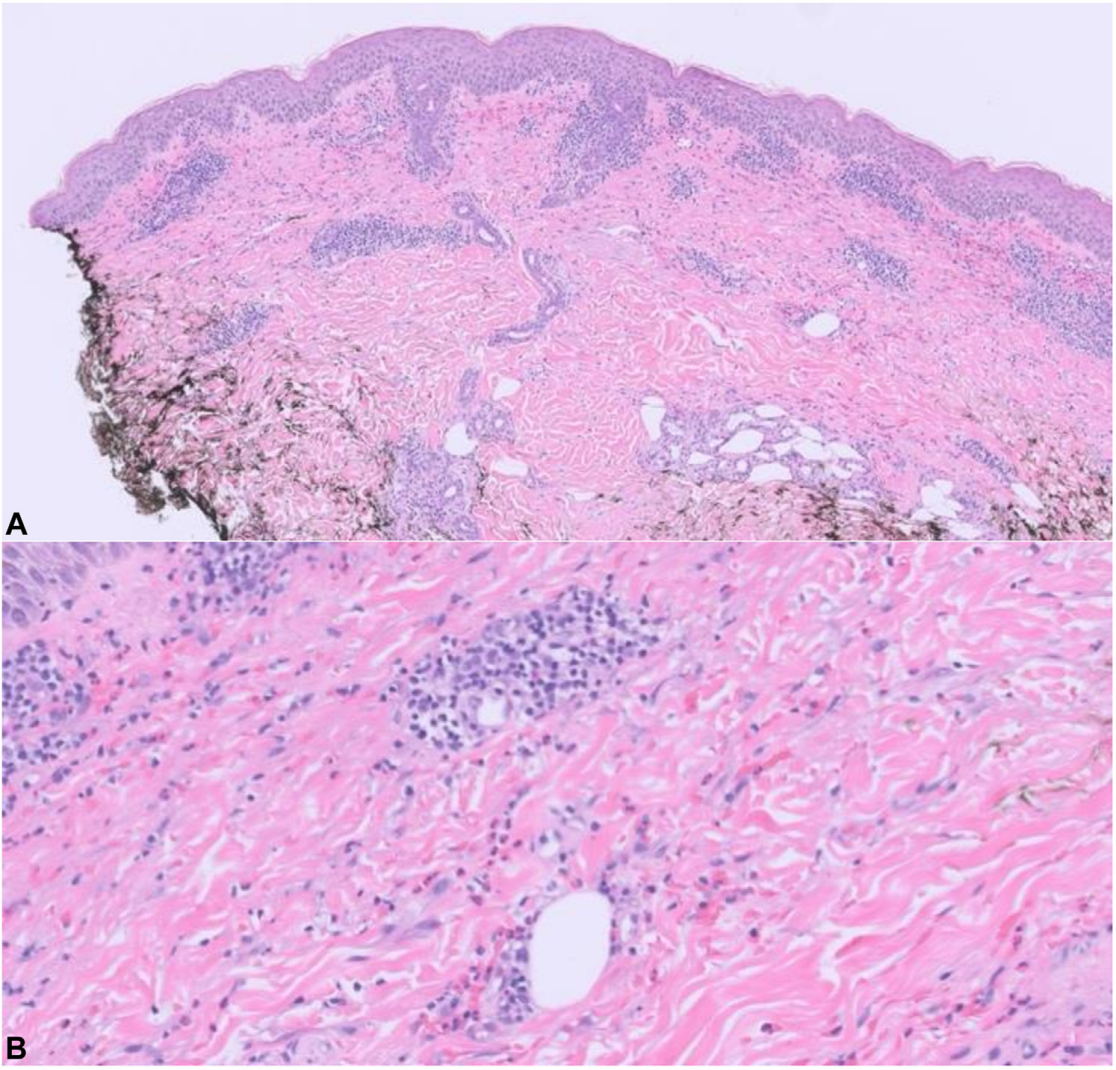
H&E of skin biopsy of abdomen from initial rash (**A**) (10× magnification) and (**B**) (40× magnification) showing superficial perivascular infiltrate and interstitial-mixed cell infiltrate comprised of lymphocytes, eosinophils, and neutrophils, consistent with drug eruption.

A 77-year-old woman with past medical history significant for hypertension, hyperlipidemia, coronary artery disease, type II diabetes mellitus, Crohn’s disease, asthma, and latent tuberculosis was discharged to an inpatient rehabilitation facility after a skin graft for a non-healing wound. While at rehab, isoniazid was resumed for her latent tuberculosis infection. Four days after medication administration she developed a full-body exanthematous rash with facial edema (Fig 1), eosinophilia, and elevated creatinine. Although the timeline from drug start to rash development was short for DRESS, the patient had previously received isoniazid for an unknown length of time. Her RegiSCAR score was 4 (Afebrile [-1]; eosinophil % = 33.6, white blood count = 23.3, absolute eosinophil count = 7829 cells/μL [+2]; skin rash extent > 50% [+1]; edema and purpura present [+1]; internal organ involvement with elevated creatinine [+1]), indicating probable DRESS. A biopsy was performed which showed superficial perivascular and interstitial mixed-cell infiltrate (Fig 2), consistent with drug eruption.

The patient was diagnosed with DRESS, started on oral steroids, and isoniazid was discontinued. Her hospital course was complicated by severe otitis externa due to *Pseudomonas aeruginosa* and *extended-spectrum beta-lactamase* *Klebsiella,* and she was started on a 10-day course of meropenem without complication. Her rash improved on systemic treatment, and she was discharged home on a prednisone taper 13 days after rash onset.

**Fig 3:**
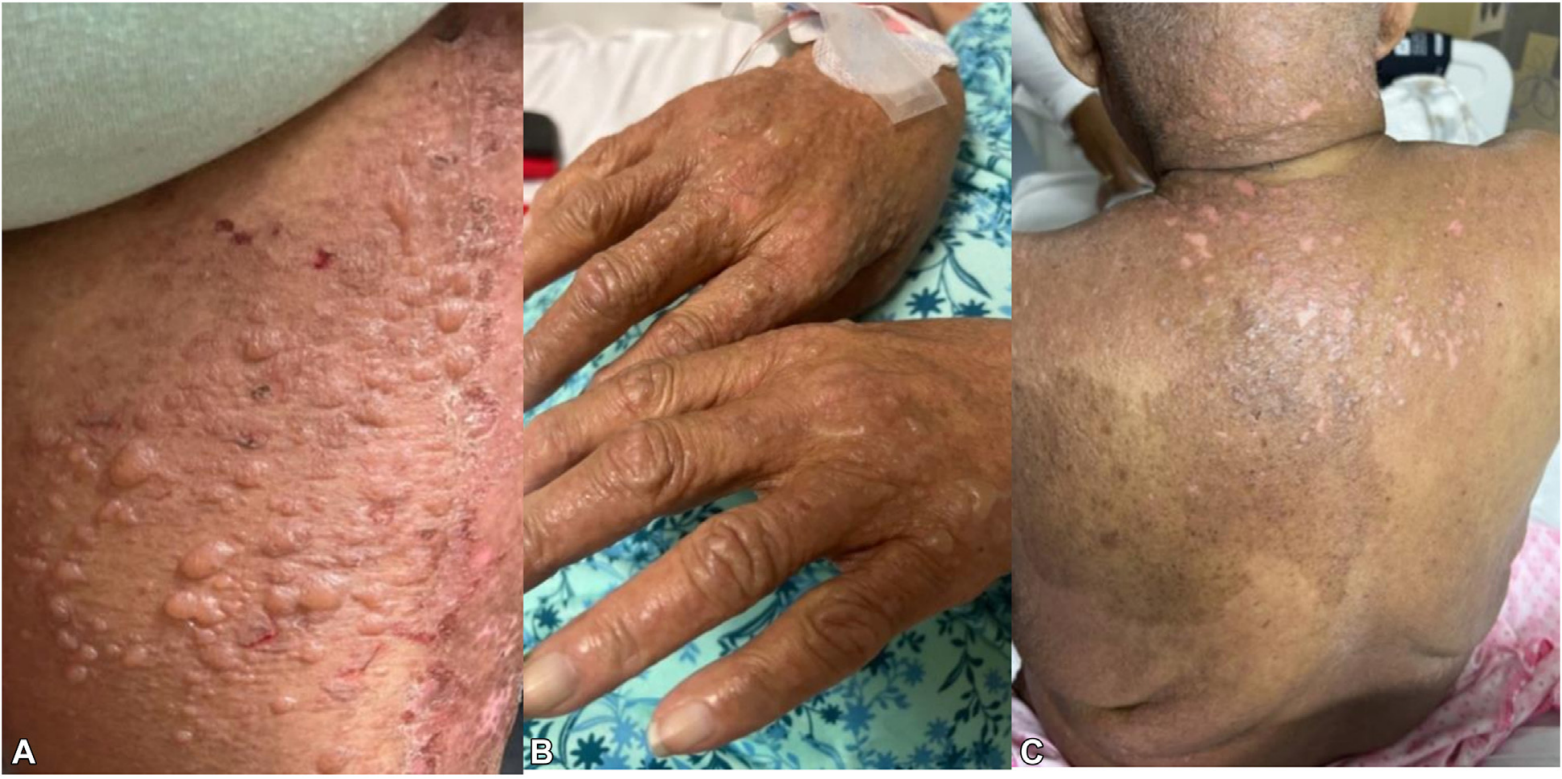
Within 2 days of reexposure to meropenem for otitis externa, the patient developed a new vesiculobullous rash involving the (**A**) left lower extremity and (**B**) dorsum of the hands. **C,** The patient’s skin began to denude on her back with light lateral pressure, indicative of positive Nikolsky sign.

The patient re-presented to the hospital 2 weeks after discharge, this time with worsening morbilliform rash and eosinophilia, concerning for flaring DRESS, and bilateral ear pain. The patient completed her steroid taper 1 week prior to re-presentation. A repeat culture of ear drainage grew *Pseudomonas* and *Candida tropicalis* and was subsequently started on meropenem and fluconazole. The following day, her rash evolved into a vesiculobullous reaction. Upon reevaluation by the dermatology service, the patient had a positive Nikolsky sign (Fig 3). Given this new finding and rash evolution, biopsies were performed for hematoxylin and eosin staining, direct immunofluorescence, and frozen sectioning.

**Fig 4:**
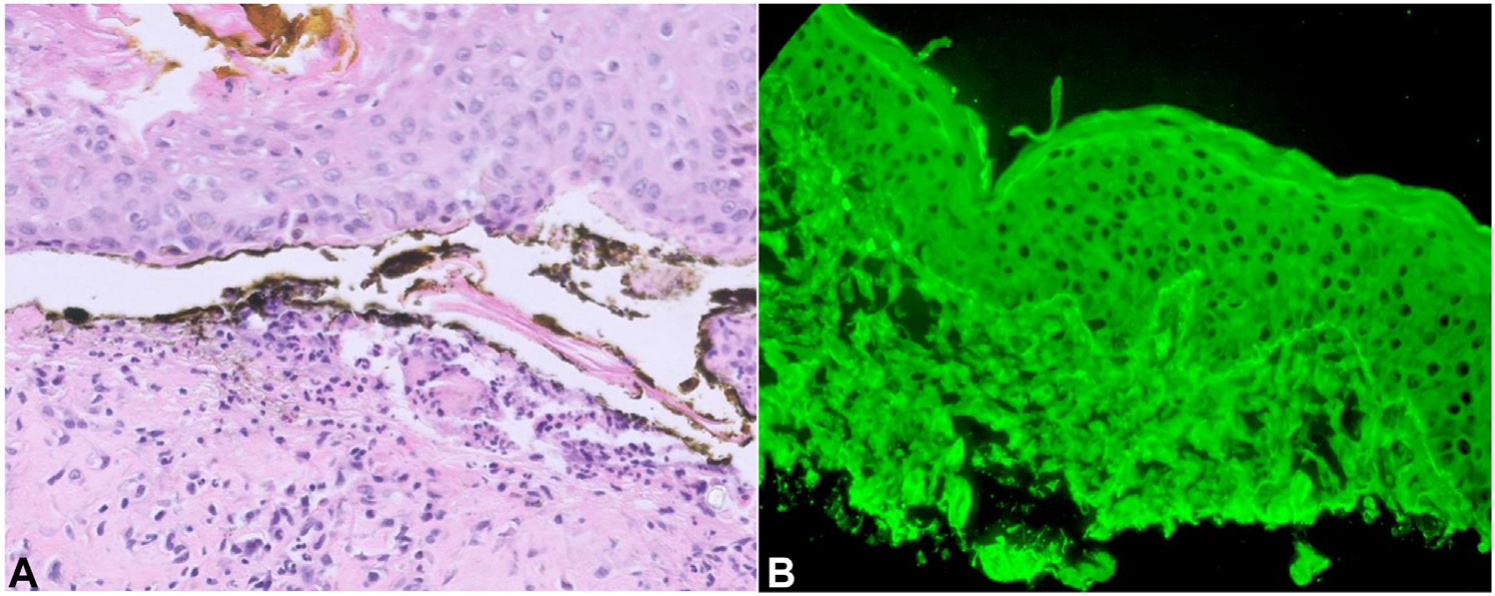
**A,** H&E of skin biopsy (40× magnification) showing neutrophil-rich subepidermal blister. **B,** Direct immunofluorescence showing linear deposition of IgA at the basement membrane zone.

H&E and frozen section of the new vesiculobullous rash demonstrated subepidermal bulla with neutrophils and associated direct immunofluorescence showed linear IgA deposition along the basement membrane zone, consistent with linear IgA bullous disease (Fig 4). The patient was started on high dose intravenous steroids and dapsone 50 mg daily. Meropenem was discontinued as the suspected drug culprit of her DILABD. After 3 days of high dose steroids and consequent improvement in her bullous disease, she was switched to oral steroids and dapsone was tapered.

## Discussion

Given the evolution of this patient’s disseminated rash and its temporal relationship with the administration of meropenem with prior sensitization by isoniazid, we propose that the developed MDH with 2 distinct clinical presentations—DRESS in the setting of isoniazid and TEN-like linear IgA bullous dermatosis in the setting of repeated exposure to meropenem.

We posit our patient was in a hypersensitive state during her repeat hospital admission for her flaring DRESS. She was initially sensitized to meropenem during her initial admission and, when reexposed to the drug with flaring DRESS, she subsequently developed a nuanced hypersensitivity reaction in the form of Stevens-Johnson syndrome/TEN-like DILABD. To date, there have been no reported cases of DILABD from meropenem in the context of flaring DRESS.

The exact mechanism of MDH remains unknown. However, the majority of MDH cases develop in patients with DRESS.^5^ Generalized drug reactions, such as DRESS, are associated with massive T-cell activation. This activation may subsequently lower the threshold of T-cell reactivity to the binding of drugs to human leukocyte antigens or T-cell receptors.^8^ These activated T cells may remain in circulation for some time and cause an ongoing predisposition to react to other drugs. The clinical consequence of this reactivity may develop into a “flare-up” reaction or may result in new additional sensitizations with unique clinical manifestations, resulting in MDH.

The risk factors of MDH are similar to DRESS. The same drugs implicated in DRESS, such as anticonvulsants, certain antibiotics, and allopurinol, are also involved in causing MDH. Drugs given in high concentration or in combination therapy, like piperacillin/tazobactam or amoxicillin/clavulanic acid, are common in drug hypersensitivity reaction; these same risk factors were observed in our patient who received a high concentration of meropenem with concomitant fluconazole. Therapies given for a longer period also put patients at risk for MDH.^5^ For our patient, she was given meropenem for 10 days during her initial episode of otitis externa, and this prolonged treatment seems to increase T-cell reactions.^5^

It is difficult to understand exactly why stimulation by 1 drug could progress to stimulation by another. Regardless, actions can be taken to possibly mitigate the risk of the development of MDH in patients with severe T-cell reactions, such as minimizing use of unnecessary drugs, avoiding antibiotics unless absolutely indicated, engaging in antibiotic stewardship based on the microorganism, and ameliorating a hypersensitive immune system with systemic treatment.

## Conflicts of interest

None disclosed.
